# Environmental Pollutants and Oxidative Stress in Terrestrial and Aquatic Organisms: Examination of the Total Picture and Implications for Human Health

**DOI:** 10.3389/fphys.2022.931386

**Published:** 2022-07-22

**Authors:** Gloria Oiyahumen Anetor, Nnenna Linda Nwobi, Godwin Osaretin Igharo, Oyebola Oluwagbemiga Sonuga, John Ibhagbemien Anetor

**Affiliations:** ^1^ Department of Human Kinetics and Health Education, National Open University of Nigeria, Abuja, Nigeria; ^2^ Department of Chemical Pathology, BenCarson School of Medicine, Babcock University, Ilishan, Nigeria; ^3^ Department of Medical Laboratory Science, School of Basic Medical Sciences, College of Medical Sciences, University of Benin, Benin, Nigeria; ^4^ Department of Chemical Pathology, College of Medicine, University of Ibadan, Ibadan, Nigeria

**Keywords:** aquatic organisms, antioxidants, chronic diseases, environmental pollution, environmental pollutants, free radicals, oxidative stress, terrestrial organisms

## Abstract

There is current great international concern about the contribution of environmental pollution to the global burden of disease particularly in the developing, low- and medium-income countries. Industrial activities, urbanization, developmental projects as well as various increased anthropogenic activities involving the improper generation, management and disposal of pollutants have rendered today’s environment highly polluted with various pollutants. These pollutants include toxic metals (lead, cadmium, mercury, arsenic), polycyclic aromatic hydrocarbons, polychlorinated biphenyls, pesticides and diesel exhaust particles most of which appear to be ubiquitous as well as have long-term environmental persistence with a wide range of toxicities such as oxidative stress among others. Oxidative stress, which may arise from increased production of damaging free radicals emanating from increased pollutant burden and depressed bioavailability of antioxidant defenses causes altered biochemical and physiological mechanisms and has been implicated in all known human pathologies most of which are chronic. Oxidative stress also affects both flora and fauna and plants are very important components of the terrestrial environment and significant contributors of nutrients for both man and animals. It is also remarkable that the aquatic environment in which sea animals and creatures are resident is also highly polluted, leading to aquatic stress that may affect the survival of the aquatic animals, sharing in the oxidative stress. These altered terrestrial and aquatic environments have an overarching effect on human health. Antioxidants neutralize the damaging free radicals thus, they play important protective roles in the onset, progression and severity of the unmitigated generation of pollutants that ultimately manifest as oxidative stress. Consequently, human health as well as that of aquatic and terrestrial organisms may be protected from environmental pollution by mitigating oxidative stress and employing the principles of nutritional medicine, essentially based on antioxidants derived mainly from plants, which serve as the panacea of the vicious state of environmental pollutants consequently, the health of the population. Understanding the total picture of oxidative stress and integrating the terrestrial and aquatic effects of environmental pollutants are central to sustainable health of the population and appear to require multi-sectoral collaborations from diverse disciplinary perspectives; basically the environmental, agricultural and health sectors.

## 1 Introduction

Environmental pollution, which has continued to be on constant rise, is one of the most serious global problems of the 21st century (Anetor et al., 2006; Anetor et al., 2008; Das and Sethi, 2022). This is of uttermost concern particularly in developing or fast industrializing countries with large chemical burden emanating from numerous increased and uncontrolled anthropogenic activities as well as intentional or unintentional release of hazardous pollutants into the environment (Liang and Yang, 2019; Gong and Kong, 2022). Additionally, poor environmental awareness and weak or unimplemented environmental policies and regulation are considered as important contributors (Nwobi et al., 2021). Some of the common environmental pollutants include toxic metals such as lead (Pb), cadmium (Cd), mercury (Hg) and arsenic (As), as well as polycyclic aromatic hydrocarbons (PAHs), polychlorinated biphenyls (PCBs), pesticides and diesel exhaust particles among others (Anetor et al., 2008; Mojiri et al., 2019; Muñoz et al., 2019). These environmental pollutants have negative impacts on both the aquatic and terrestrial environments such as the air, soil and water bodies, affect the sustainability of the inhabiting organisms, and inadvertently alter the natural ecosystem with negative implications for human health (Akram et al., 2019; Zaghloul et al., 2020; Tsai et al., 2021).

The growing concern about the deleterious effects of environmental pollution on all living organisms including human health, has informed the formation of the Global Burden of Disease–Pollution and Health Initiative (GBD-PHI) (Landrigan et al., 2018; Hu, 2019). This concern is greater in the low-income and middle-income countries where the major attention continues to focus on infectious and communicable diseases (Coker and Kizito, 2018; Landrigan et al., 2018; Odo et al., 2022). However, a recent report of analysis on global burden of disease modeling has revealed that low-income countries in Sub-Saharan Africa have the highest disease-burden and early death attributable to environmental pollution (Coker and Kizito, 2018; Landrigan et al., 2018; Odo et al., 2022).

Several reports have revealed that exposure of organisms to environmental pollution contributes to oxidative stress: a state of imbalance between the rate of generation of very damaging free radicals such as reactive oxygen species (ROS), and neutralizing molecules which are mostly nutritional factors called antioxidants, in favour of ROS (Sharifi-Rad et al., 2020; Bello-Medina et al., 2022). Oxidative stress if uncontrolled, may alter distinct biochemical, cellular and physiological mechanisms which may directly or indirectly affect the survival of the aquatic and the terrestrial organisms with an increased propensity to cause several human diseases most of which may be chronic (Xu et al., 2018; Cao et al., 2020; Zheng et al., 2021; Sandys and Te-Velde, 2022). This in turn, could have diverse detrimental effects on human health with far reaching impacts on the population, community and society at large. However, several reports have shown that the health of the ecosystem and that of the organisms therein particularly human health, may be safe guarded in the face of environmental pollution by mitigating oxidative stress as well as employing the principles of nutritional medicine essentially rooted on the antioxidant hypothesis (Halliwell and Gutteridge, 2015; Nwobi et al., 2020).

This report therefore, attempts to draw the attention of the scientific community to the enormity of environmental pollution, attendant oxidative stress and the terrestrial and aquatic contribution as well as far reaching consequences for fauna, flora and human health. These components will be examined in all perspectives in order to provide a total picture as well as emphasizing the need to treat them as inseparable and not in isolation.

## 2 Environmental Pollution

**FIGURE 1:**
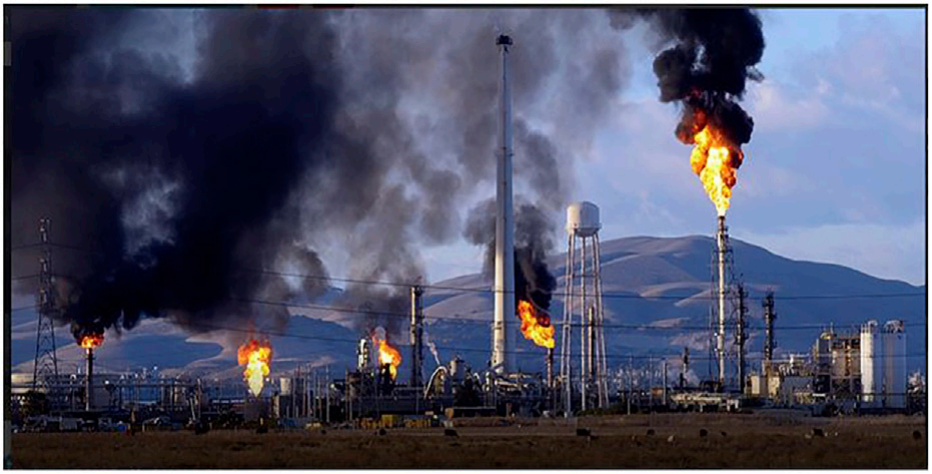
Typical pollution of the environment from a refinery (https://africanclimatereporter.com/2019/06/05/environment-day-climatologist-tasked-industry-company-refinery-owners-in african-on-mandatory-trees-planting-and-reduce-polluting-the-atmosphere/).

Environmental pollution has been described as the slow and insidious process of destroying the earth by contaminating it with pollutants thereby, killing its ability to support life (Vesilind et al., 2013; Seiyaboh and Izah, 2019). Most environmental pollutants appear to be ubiquitous as well as have long-term persistence in the aquatic and terrestrial environments with a wide range of toxicities to the inhabiting organisms as well as diverse human health risks. Increased environmental pollution has been linked substantially to increased industrialization (Figure 1), urbanization and developmental projects as well as various increased anthropogenic activities which involve the improper use, control, management and disposal of these pollutants and products that generate them (Beckers and Rinklebe, 2017; Obeng-Gyasi, 2019; Raju, 2022).

The major concerns regarding environmental pollution are basically human health and welfare, the health of other aquatic and terrestrial organisms as well as the preservation of the ecosystem and nature in general. According to Vesilind et al. (2013), “human health and well-being can be impacted by environmental pollution in two distinct ways; 1. On a personal level of detrimental health effect through contaminated water, air or food. 2. On a global level as a slow yet progressive deteriorations of aquatic and terrestrial habitats and the organisms therein, resulting in the eventual destruction of human species and perhaps, all lives”.

Some of the common environmental pollutants include toxic metals such as lead (Pb), cadmium (Cd), mercury(Hg) and arsenic (As), as well as polycyclic aromatic hydrocarbons (PAHs), polychlorinated biphenyls (PCBs), pesticides and diesel exhaust particles among others (Anetor et al., 2008; Mojiri et al., 2019). Some of the common exposure sources of these pollutants and their health effects are listed in Table 1.

**TABLE 1:** Major environmental pollutants, sources and associated health effects.

Environmental pollutant	Exposure sources	Selected health effects	Selected References
Toxic metals			
Lead	Industrial emissions, electronic waste, batteries, traditional medicines, ammunitions, glazed ceramics, lead contaminated water, paint, dust, varieties of industrial products e.g toys	Nervous system disorders (slow nerve conduction, fatigue, mood swings, drowsiness, reduced intelligence quotient), Circulatory system disorders (hypertension, impaired haemopoeisis). Gastrointestinal system disorders (colic/pain, nausea, vomiting, diarrhea, constipation), and hormonal disorders (infertility, decreased libido), cancer and bone disorders among others	Rehman et al., (2018); Steenland et al., (2019); Obeng-Gyasi, (2019); Pain et al., (2019); Nwobi et al., (2019), Charkiewicz and Backstrand, (2020); Nwobi et al., (2021)
Cadmium	Food grown in cadmium contaminated soil, phosphate fertilizers, tobacco smoke, inhaling aerosols from industrial products such as steel, plastics and nickel–cadmium batteries.Cd in the air due to smelting, soldering, welding and refining metals containing Cd	Renal and hepatic dysfunction, pulmonary edema, testicular damage, osteomalacia, damage to the adrenals and hemopoietic system; coronary heart disease, stroke, peripheral artery disease and various cancers	Mortensen et al., (2011); Tinkov et al., (2018); Mezynska and Brzóska, (2018); Niño-Savala et al., (2019); Genchi et al., (2020)
Mercury	Natural sources such as soil, forests, lakes, open oceans, volcanic emissions, mining activities, wastes from combustion, urban and industrial discharges, as well as dental amalgams in medicine	Various health abnormalities related to genetic, reproductive, renal, neurological, and cardio-vascular systems	Andreoli and Sprovieri, (2017); Basu et al., (2018); Selin, (2009); Xu et al., (2018); Ali et al., (2019)
Arsenic	Mining, smelting of metal ores, waste emissions, residuals from water treatment, medical waste, burning of fossil fuels, use of arsenic-containing pesticides, herbicides, insecticide, fertilizers, timber and wood preservatives	Induces various cancers, damages the stomach, kidneys, liver, heart and nervous system. Affects the lungs leading to shortness of breath, chest pain, and Cough and in severe cases, may cause death	ATSDR, (2016); Rahaman et al., (2021); Bundschuh et al., (2021)
Polycyclic aromatic hydrocarbons (PAHs)	Incomplete combustion of carbonaceous materials during energy and industrial production process, burning of natural compounds such as wood, coal, gasoline, diesel and tobacco smoke	Respiratory problems, acute bronchitis, heart problems, lung cancer, ovarian tumor and primary ovarian insufficiency aggravation of preexisting heart and lung disease and asthma	Ravindra et al., (2008); Sumanasekera et al., (2018); Mojiri et al., (2019); Patel et al., (2020); Ali et al., (2021); Priya et al., (2021)
Polychlorinated biphenyls (PCBs)	Petrochemicals; leaks of lubricating fluids from vehicles and machines, leaks of coolant and insulation fluid from damaged heat exchangers and transformers and from waste disposal sites	Impairs sexual development, disrupts reproductive function, causes developmental toxicity, immunotoxicity, metabolic diseases such as type II diabetes and thyroid disorders	Catalani et al., (2019); Djordjevic et al., (2020); Mikolajczyk et al., (2020); He et al., (2021)

### 2.1 Effects of Environmental Pollution on Terrestrial and Aquatic Organisms; a Vicious Cycle

**FIGURE 2:**
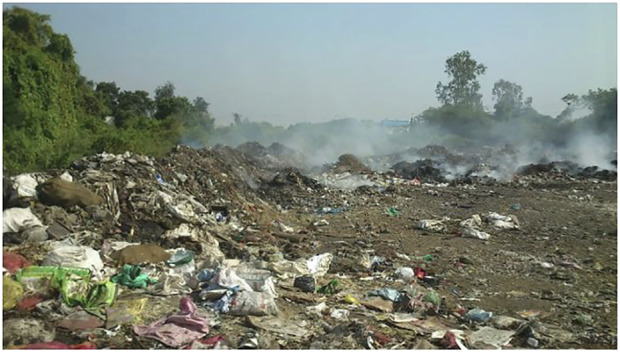
The Effect of Soil Pollution on the living (Baruah, 2020).

**FIGURE 3:**
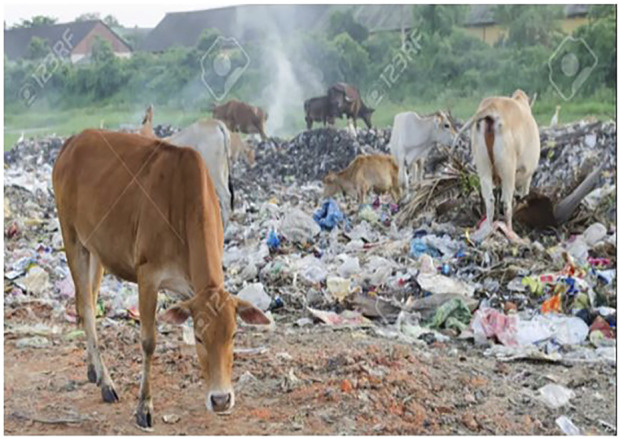
Animals in a Polluted environment grazing on polluted soil(https://www.123rf.com/photo_29,849,393_cattle-and-land-pollution.html).

Environmental pollutants from various sources such as air, water and soil, have direct and indirect adverse impacts on aquatic and terrestrial environments as well as the inhabiting organisms. The air pollutants after generating varying degrees of physiological alterations in humans, animals and birds, inadvertently settle on soil and water sources. The contaminated soil looses its fertility, texture and structure, which impacts negatively on plants as they absorb nutrients and water from this contaminated soil for their survival and this ultimately affects the food chain. The soil is also made inhabitable for the organisms that naturally dwell in it (Figure 2). Animals in a polluted environment are not spared from the toxic exposure as they graze on the polluted soil (Figure 3).

**FIGURE 4:**
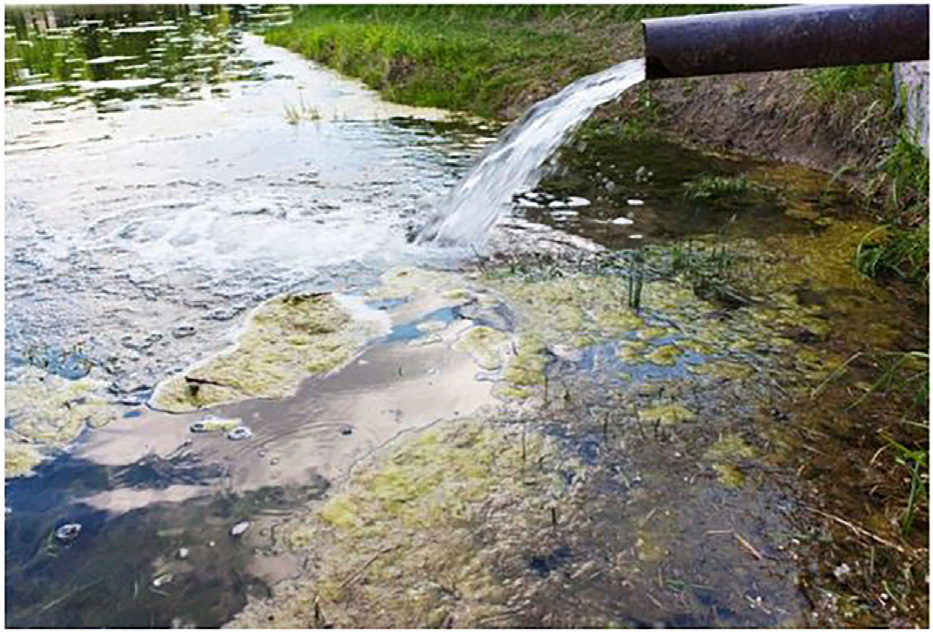
Water pollution (https://www.openaccessgovernment.org/water-company-fined-for-unpermitted-Pollution/48105/).

Contaminants in the soil can also directly enter the human system through contact with the skin or inhalation of polluted soil or dust. In the same vain, water pollution has a negative impact on the viability and sustainability of water resources, as well as the proper growth and development of fish and other aquatic organisms, which are considered as important food sources (Figure 4). These all lead to a state of vicious cycle. Animals consume the contaminated fish and plants as well as drink the contaminated water leading to serious threat to their health and sustainability as well as disruption in the predator–prey interactions. The pollutants continue to travel up the food chain until they get to humans who eat the food and experience deleterious effects on their health as well their behavior and productivity (Chang et al., 2019; Warra and Prasad, 2020). This was classically manifested in the well-known Minamata disease; an environmental epidemic in Minamata Bay, Japan, caused by methyl-mercury poisoning by residents who ate a considerable amount of fish contaminated with wastewater discharged from a chemical company (Murata and Karita, 2022).

### 2.2 Environmental Pollution and Flora Decline

The deleterious effects of environmental pollution are not only on human health, rather on all living things (Priyadarshanee et al., 2022). The reported forest decline in the United States and Europe for a number of tree species illustrates the effects of environmental disorder on living things other than humans (Gawel et al., 1996). Atmospheric pollutants from industrial sources such as oxidants and heavy metals could be at least partly responsible since their deposition patterns were correlated with forest decline (Grill, 1987).

Phytochelatins are oligomers of glutathione found in plants, fungi, nematodes and all kinds of algae including cyanobacteria, which serve as chelators and are thus, vital for heavy metal detoxification. The concentrations of phytochelatins have been used to show that metals are indeed most likely to be a contributing factor in the decline of forests in the northern United States considering their role specific indicators of metal stress (Grill et al., 1988; Schat and Kalff, 1992; Maresca et al., 2022). Phytochelatin concentrations in red spruce, a species in decline are higher than that of balsam fir, a species, that is, not (Gawel et al., 1996). Concentrations of phytochelatins increase with altitude, as does forest decline. They also increase across the region in forest stands that show increasing levels of tree damage. The most likely explanation of all available data on the pattern of phytochelatins concentrations and the pattern in tree damage according to species elevation and geographic distribution, is that heavy metals are a contributing cause of forest decline in northern United States (Madamanchi et al., 1991; Devos et al., 1992).

### 2.3 Environmental Pollution and Endocrine Disruptors

One of the stunning findings of the profound effects of environmental chemicals on the ecosystem about three decades ago, was the incompletely elucidated situation of endocrine disruptive substances. Endocrine disruptive substances, whose concentrations are magnified through bioaccumulation, interfere with the synthesis, secretion, transport, binding and action or elimination of natural hormones such as reproductive hormones (Sirohi et al., 2021). Thus, they have their greatest impact on reproduction not only in humans but also in birds, animals and fish (Patisaul, 2021; Robaire et al., 2022). More recent reports confirm these observations and clinical cases are becoming more evident (Rahman et al., 2018; You and Song, 2021; Reis et al., 2022).

### 2.4 The Contribution of Environmental Pollution to Reproductive Dysfunction and Developmental Events: A Threat to Species Survival

An intact physiological system is an important requirement for proper reproduction and development in humans, animals and even plants. One of the key adverse effects of environmental pollution is alteration of reproductive physiology through a number of mechanisms including oxidative stress (Ferrante et al., 2022). Reproductive organs are essential not only for the life of an organism but also for the survival and development of the species (Massányi et al., 2020). The response of reproductive organs to toxic substances differs from that of other target organs. As such, they may serve as an ideal “barometer” for the deleterious effects of environmental pollution on animal and human health (Massányi et al., 2020). It has been quite evident that our environment has been greatly polluted with resultant steadily increasing reproductive abnormalities such as infertility, decreased libido, low sperm counts as well as cancers of the reproductive tract (Canipari et al., 2020).

Persistent environmental pollutants such as PCBs exert a variety of toxic effects in animals, including disturbances of sexual development and reproductive function (He et al., 2021). Reports have shown that ovarian cells exposed to different concentrations of PAHs resulted in ovarian tumor growth and primary ovarian insufficiency (Sumanasekera et al., 2018; Priya et al., 2021). The effects of cadmium, lead, or mercury on the structure and function of reproductive organs have been reviewed in Massányi et al., 2020. The review indicated that the testis and ovary are particularly sensitive to cadmium, lead, and mercury because these organs are characterized by an intense cellular activity, where vital processes of spermatogenesis, oogenesis, and folliculogenesis occur (Massányi et al., 2020). In ovaries, manifestation of toxicity induced by cadmium, lead, or mercury includes decreased follicular growth, occurrence of follicular atresia, degeneration of the corpus luteum and alterations in cycle. In the testes, toxic effects following exposure to cadmium, lead or mercury includes alterations of seminiferous tubules, testicular stroma, decrease of spermatozoa count, motility and viability as well as aberrant spermatozoa morphology (Massányi et al., 2020). Our studies in this environment on mothers and newborns revealed a disproportionate number of babies with low birth weight, decreased length and reduced head circumference (Ikeh-Tawari et al., 2013). Damaging effects of environmental chemicals on reproductive capacity of flora is also well known (Tripathy et al., 2022).

## 3 The Concept of Oxidative Stress and its Pathophysiology

Free radicals such as reactive oxygen species (ROS) are extremely reactive and unstable species that are constantly produced because of redox chain reactions or as metabolic byproducts. Under normal physiological conditions and antioxidant concentrations, ROS influence normal physiological functions and are considered as major signaling messengers involved in maintaining cellular homeostasis such as cellular metabolism, growth, development and programed cell death (Niki and Noguchi, 2021). As a result, in natural conditions, the normal healthy cell maintains a dynamic equilibrium between ROS overproduction and detoxification via antioxidant mechanisms.

However, uncontrolled ROS actions, resulting from excessive ROS generation that overcomes the cellular antioxidant defense mechanisms or by alteration in the functioning of the antioxidant defense system leads to oxidative stress.

Though oxidative stress is a phenomenon most scientists in the biomedical community are reasonably familiar with, one of the simplest available explanations of oxidative stress is the legend from Kelly (2003): ‘Oxidative stress exists when there is an excess of free radicals over antioxidant defences. As a consequence, free radicals attack and oxidize other cell components such as lipids, proteins and nucleic acids resulting in tissue injury and in some cases, the influx of inflammatory cells to the site of injury’ (Kelly, 2003). Simply put, oxidative stress may arise from several avenues such as increased production of damaging reactive species emanating from increased pollutant burden as well as absence or depressed bioavailability of antioxidant defenses.

**FIGURE 5:**
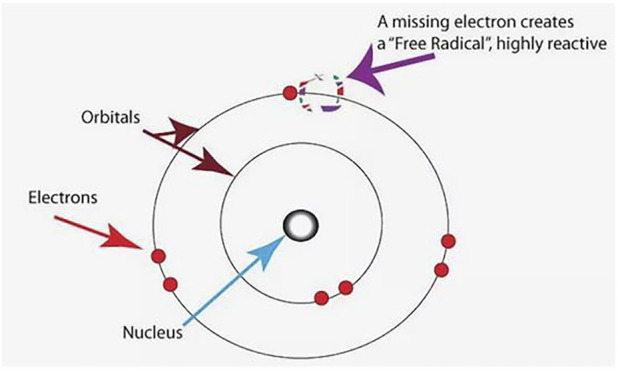
Free radical showing an unpaired electron (Helmenstine, 2020).

The significance of oxidative stress mainly rests in the unstable and reactive nature of molecules involved. Unlike in normal situations when the electrons exist in pairs spinning in opposite directions, free radicals have unpaired electrons in their outer orbitals, which render them extremely reactive (Figure 5). Free radicals appear to extract electrons from a neighboring molecule thus inactivating the molecule. These Free radicals, irrespective of the sources, are potentially dangerous and indiscriminately damaging to biological molecules in both terrestrial and aquatic habitats. If the resulting damage is extensive, this may culminate in cellular damage and attendant organ dysfunction. However, the damaging cascade of events can be halted or blocked by antioxidants, through a process of neutralization, resulting in innocuous and non-toxic products.

The magnitude of damage may be contingent upon the bioavailability of scavenging antioxidant molecules as well as the inverse relationship between free radicals and antioxidant capacity. Damage from aberrant reactivity of free radicals could be a combination of a raised environmental derived free radical burden and antioxidant deficit. It should however be borne in mind that low-level of oxidative stress may be beneficial to the organism especially in signaling in the immune system (Pizzino et al., 2017; Flohé, 2020; Margaritelis et al., 2022).

The ensuing or resultant increased oxidative stress can cause oxidative damage to important biomolecules within the cells, which could cause protein oxidation, cellular DNA damage, electrolyte leakage with cell membrane lipid peroxidation and eventually apoptotic cell death and associated numerous pathologic states. A body of evidence has suggested that oxidative stress operates through multiple mechanisms to adversely affect a number of target organs and systems leading to various disease states. Such diseases include placental diseases (Ruano et al., 2022), atherosclerosis, chronic obstructive pulmonary disease, Alzheimer’s disease, and cancer (Forman and Zhang, 2021). It is also worthy of note that the degree to which oxidative stress participates in the pathology of diseases varies greatly, such that the effectiveness of increasing antioxidant defence may be limited in some diseases (Forman and Zhang, 2021).

### 3.1 Oxidative Stress; a Pathophysiological Response to Environmental Pollution

Rachel Carson in her book “Silent Spring”, was the first to draw the attention of the global scientific community to the damaging effects of indiscriminate production, poor handling and disposal of chemicals as well as the attendant deleterious effects on the ecosystem or human health through pathophysiological pathways of altered biochemical and physiological mechanisms (Carson, 2002). Sideris and Moore (2008) attempted to address this in their examination of Carson’s contribution to ensuring a safer healthier environment. They brought to the fore that Silent Spring was a pivotal catalyst to creating widespread environmental legislation of air and water pollution and endangered species in the terrestrial and aquatic environments. This created the desired public awareness about environmental disorders emanating from chemical pollution. In Silent Spring, Rachel Carson endeavored to bridge the existing disconnection among scientists, laypeople, governments and corporations and based on her eye-opening contribution has been described as “The Right Person, at the Right Time, with the Right Message” (Coffman, 2018). It is perhaps time the existence of oxidative stress is considered a gap or challenge which Rachel Carson left for us to fill. This might also be one of the omissions now considered a collateral damage to environment molded life and represents a response to environmental pollution (Souder, 2012).

Global advances in research using diverse approaches have revealed that oxidative stress is currently recognized as a unifying feature underlying toxic events associated with environmental pollution (Leni et al., 2020; Paithankar et al., 2021; Traina et al., 2022). Virtually all the environmental chemicals in the aquatic or terrestrial environment, elicit oxidative stress, which constitutes a major risk or hazards to inhabitants of polluted environment and which in the absence of appropriate protection mechanisms such as available antioxidant defense system can lead to physiological and biochemical derangement that end up in disease (Nwobi et al., 2020). Increased oxidative stress is widely recognized as a significant factor in the pathogenesis of a number of chronic diseases caused by environmental pollution. Perhaps in discussing environmental pollution, the associated oxidative stress and attendant altered physiology as a final pathway through which the adverse consequences of pollution are exerted, is borne in mind and given the level of proper consideration. Pockets of evidence also indicate that the sensitivity to pollution may be related at least in part, to the available antioxidant defense mechanism at the target site (Poljšak and Fink, 2014; Eftekhari et al., 2018; Nwobi et al., 2020).

## 4 Antioxidant Defence Mechanisms: Antidotal Rescue for Polluted Ecosystems

The antioxidant defense mechanisms occur in enzymatic and non-enzymatic forms. Enzymatic antioxidants, which are also known as natural antioxidants or endogenous antioxidants, include superoxide dismutase (SOD), catalase (CAT), glutathione peroxidase (GPx), glutathione reductase (GR) and Glutathione S-transferase (GST) (Irato and Santovito, 2021). On the other hand, the non-enzymatic antioxidants, also known as exogenous antioxidants, are mostly obtained from dietary fruits and vegetables and include micronutrients such as β-carotene (vitamin A), vitamin C (Ascorbic acid), vitamin E (α tocopherol), zinc (Zn) and selenium (Se) (Lindshield, 2019). The production of antioxidants has direct and indirect link to the environment and may indeed be a rescue to inhabitants of polluted environment engulfed by oxidative stress.

### 4.1 Enzymatic Antioxidants

#### 4.1.1 Superoxide Dismutase, Catalase and Glutathione Peroxidase

Superoxide dismutase, catalase and glutathione peroxidase are enzymes that represent the first line of antioxidant defence and are functionally interconnected because the product of the reaction catalyzed by SOD, hydrogen peroxide (H_2_O_2_), is the substrate of both CAT and GPx. (Ighodaro and Akinloye, 2018; Irato and Santovito, 2021). Superoxide dismutases are metalloenzymes which are found in the cytosol and the mitochondrial intermembrane (Copper, Zinc-SOD), the mitochondrial matrix and inner membrane (Manganesse-SOD), and extracellular compartment (Copper, Zinc-SOD) (Rosa et al., 2021). Superoxide dismutases remove oxy-radicals by converting the superoxide anion free radical (*•*O2*−*) to hydrogen peroxide (H_2_O_2_) and molecular oxygen (O_2_) (Rosa et al., 2021).

Catalase converts H_2_O_2_ to less toxic gaseous oxygen O_2_ and water (Fujiki and Bassik, 2021). On the other hand, GPx transforms H_2_O_2_ to O_2_ and water (Ighodaro and Akinloye, 2018; Irato and Santovito, 2021). Furthermore, GPx promotes the conversion of peroxide radicals to alcohol by oxidizing the reduced glutathione (GSH) to oxidized glutathione (GSSG) (Ighodaro and Akinloye, 2018).

#### 4.1.2 Glutathione Reductase and Glutathione S-transferase

Glutathione reductase promotes the conversion of GSSG to GSH in order to replenish the pool of GSH in the living cells (Radwan et al., 2020). While GSTs are phase II enzymes that are involved in the conjugation of electrophilic components to glutathione and in the protection from oxidative effects and peroxidative products of lipids or DNA (Strange et al., 2001).

### 4.2 Non-enzymatic Antioxidants

#### 4.2.1 Vitamin C (Ascorbic Acid)

Vitamin C is a water-soluble micronutrient ,that is, abundant in natural rich sources such as fresh fruits and green leafy vegetables (Devaki and Raveendran, 2017). Vitamin C is a powerful reducing agent and a broad-spectrum antioxidant that fights a wide range of free radicals, including ROS (Malik et al., 2021). Vitamin C is involved in the first line of antioxidant defense acting as an excellent donor of electrons for free radicals that need electrons to regain their stability (Zhitkovich, 2021). As a result, vitamin C quenches their reactivity and shields the cellular components from free radical-induced cellular damage. Vitamin C also aids in the regeneration of the antioxidant form of vitamin E by decreasing the tocopheroxyl radicals (Shakeri et al., 2020). Vitamin C comes from plant, thus if plants are not protected from the ravages of oxidative stress, the biochemical and physiological roles of this pivotal antioxidant may be abrogated with wide range health consequences.

#### 4.2.2 Vitamin E

Vitamin E (tocopherol) is a lipid soluble micronutrient. Natural forms of vitamin E can be obtained from plant seeds, fruits, vegetables and nuts (Chun et al., 2006). All vitamin E forms are potent antioxidants because they possess similar phenolic moieties; they scavenge lipid peroxyl radicals by donating hydrogen from the phenolic group on the chromanol ring (Jiang, 2014; Niki and Noguchi, 2021). Vitamin E functions as a chain-breaking antioxidant that protects cellular membranes against ROS (Traber and Jeffrey, 2007). If plants are not protected from the effects of oxidative stress, the biochemical and physiological functions of this important antioxidant may be compromised, resulting in a variety of health problems.

#### 4.2.3 Beta-Carotene (Provitamin A)

Beta-carotene is a fat-soluble natural pigment primarily found in plants where it is abundant in orange and yellow fruits such as carrots and mangoes as well as green leafy vegetables such as pumpkins (Durante et al., 2014; Rodriguez-Amaya, 2016). Beta-carotene is enzymatically cleaved in the intestinal mucosa by beta-carotene 15,15′-monooxygenase at the central double bond to form two molecules of vitamin A (retinol), which is involved in antioxidant defence (Álvarez et al., 2014; Olza et al., 2017). Beta-carotene acts as antioxidant by effectively neutralising ROS by reducing their propagation, quenching singlet oxygen and preventing cellular damage thereby, decreasing oxidative stress and oxidative damage to DNA (Bahonar et al., 2017; Chen et al., 2021).

#### 4.2.4 Selenium

Selenium is another micronutrient whose availability in foods depends largely on Se content in the soil where plants grow (Hu et al., 2021). Nuts, cereals, grains, cruciferous vegetables, garlic, onions as well as eggs, fish, meat and meat products are very rich in Se (Hu et al., 2021). Selenium is involved in antioxidant function (Burk and Hill, 2015). As a fundamental part of GPX and other enzymes, the main antioxidant role of Se is due to the activities of the seleno-enzymes and avoidance toxicity by selenoproteins (Gaetke, 2003; Burk and Hill, 2015).

#### 4.2.5 Zinc

Zinc is an essential micronutrient that has richest food sources as oysters and meat such as beef, veal, pork and lamb (Jun and Betts, 2000). Zinc has antioxidant properties that are based on a variety of mechanisms (Prasad, 2014). Zinc is a co-factor of SOD that catalyzes the dismutation of ˙O⁻2 to H_2_O_2_. It is also an inhibitor of nicotinamide adenine dinucleotide phosphate (NADPH) oxidase - a group of plasma membrane-associated enzymes which catalyze the production of O⁻2 from oxygen by utilizing NADPH as electron donor, resulting in decreased generation of ROS (Prasad, 2014). Zinc competes with Fe++ and Cu++ ions for binding to cell membranes and protein, displacing these redox active metals that catalyze the formation of OH from H_2_O_2_. Zinc also promotes the production of metallothionein, a cysteine-rich protein, that is, an effective ˙OH scavenger. Zinc protects bio-molecules from oxidation by binding to their sulfhydryl groups, promotes the activation of antioxidant proteins, molecules and enzymes such as GSH, CAT, and SOD, while inhibiting the activity of oxidant-promoting enzymes such inducible nitric acid synthase and NADPH oxidase (Prasad, 2014). Zinc regulates the activity of nuclear factor erythroid 2-related factor 2 (Nrf2); a key transcription factor that controls the gene expression of antioxidant proteins and enzymes such as GSH and SOD, as well as detoxifying enzymes such as GST, by binding to an antioxidant responsive element in the promoter region of the target gene (Prasad, 2014). Thus, up-regulating Nrf2 activity and down-regulating the generation of ROS (Prasad, 2014).

## 5 Implications of Environmental Pollution for Human Health

### 5.1 Effects of Air Pollution

Environmental pollution from various sources such as air, water and soil, has an overarching effect on human health. Several studies have discovered a strong link between air pollutants and respiratory diseases, chronic obstructive pulmonary disease, asthma, bronchitis symptoms and lung cancer (Nhung et al., 2017; Almetwally et al., 2020). Air pollutants have also been associated to cardiovascular diseases such as heart failure, myocardial infarction, strokes and arrhythmias as well as increased mortality (Shah et al., 2013; Cai et al., 2016; Almetwally et al., 2020). Exposure to air pollutants has been linked to type 2 diabetes (Janghorbani et al., 2014), gestational diabetes mellitus (Zhang et al., 2020), as well as increased chances of hospitalization of children that are epileptic (Cakmak et al., 2010; Cui et al., 2017; Bao et al., 2019).

### 5.2 Effects of Water Pollution

The leading causes of water pollution include anthropogenic sources such as untreated industrial effluents, inappropriate waste disposal and agricultural run-offs. Consuming polluted water poses a significant health risk such as high incidence of water-borne infections leading to the death of the organism. For example, contaminating water supplies with pesticides has deleterious impacts on ecosystems as well as humans as these pesticides act as possible mutagens causing DNA alterations (Hassaan et al., 2020). About 1,000,000 people are poisoned acutely because of pesticide exposure with a death rate of 0.4–1.9% per annum (Eddleston, 2020; Hassaan et al., 2020; Jia et al., 2020). About 70% of these deaths could be attributed to pesticide exposure with pathophysiological mechanism at least in part involving oxidative stress. Paraquat, a hazardous fast-acting herbicide, is a notable example (Chang et al., 2022). Furthermore, long-term exposure to lower pesticide concentrations has been linked to syndromes encompassing various cancers and neurological disorders (Owens et al., 2010; Bertero et al., 2020).

### 5.3 Effects of Soil Pollution

Soil offers a foundation for plants to grow. Soil pollutants such as toxic metals, have a tendency to bio-accumulate in plants tissues altering their normal physiology and growth with negative consequences on the animals and humans who rely on them for food (Seiyaboh and Izah, 2019). As a result, there is a considerable risk of soil pollutants entering the food chain as toxins. Food toxins can enter the human body and cause a variety of disorders affecting the reproductive, respiratory, neurological, and digestive systems, as well as poisoning of organs such as the liver and kidney (Seiyaboh and Izah, 2019).

## 6 Nutrigenomics: The Promise and Application in Environmental Pollution

Nutrigenomics refers to the study of the impact of specific nutrients, dietary components or entire diet on gene expression and gene regulation (Patil et al., 2022). Although many perspectives of molecular basis of chronic diseases such as metabolomics, nutrigenomics, transcriptomics are emerging and being pursued, nutrigenomics is currently the most important and widely applied (Rodrigues-Costa et al., 2021; Patil et al., 2022).

The fundamental concepts of the field of nutrigenomics are that the progression from a healthy phenotype to a chronic disease phenotype must occur by charges in genes expression or by differences in activities of proteins and enzymes and that dietary chemicals (nutrients) directly or indirectly regulate the expression of genomic information (Kaput and Rodriquez, 2004; Irimie et al., 2019). Alteration in dietary chemicals from environmental or ecological disturbances can therefore produce altered expression of genomic events, which can lead to a form of chronic disease phenotype.

Nutrigenomics appears to be promising as a significant improvement in the understanding of the molecular basis of chronic diseases including those arising from toxic metals or toxic chemical syndrome from environmental contamination. Owing to the role micronutrients play in gene expression, they are crucially important in this case and vital for the application of this emerging science and technology (O’Rourke et al., 2020; Saito et al., 2022). Nutrigenomics is closely connected to nutrigenetics, which is an area more concerned with the investigation of how genetic variability affects body response to dietary components (Alagawany et al., 2022). Nutrigenomics is still an evolving area with a lot of promise in toxicogenomics, environmental toxicology and ecotoxicology.

## 7 Partnership Among Health, Environmental and Agricultural Agencies–A Worthwhile Venture

According to the report of Shakman (1974); one of the early environmentalists, humans currently live in an environment in which many kinds of pollution and ecological disorders are serious problems and appropriate food supplies are inadequate. Although He emphasized the consequences of environmental pollution and the ameliorative influence of nutritional factors, which are essentially antioxidant factors, he did not envisage the full dimension of the environmental monster-climate change from unbaiting environmental pollution, that man could be confronted with (Shakman, 1974; Zandalinas et al., 2021).

A growing concept of health considers health as an essential component of sustainable development, which can only be achieved through joint efforts by several sectors (World Health Organization, 1997; Anetor et al., 2006). A closer partnership between the health, environmental agencies and the agricultural sector is required to reduce the threat resulting from environmental and ecological derangements from aquatic and terrestrial environmental pollution. In contemporary world which may be considered as a chemical habitat, chemicals including metals, pesticides and PAHs are pervasive, causing a spectrum of toxicity. In the more severe cases of heavy pollutant toxicity, treatment could be nutritional, which works slowly but efficiently and safely.

In this new century, it has become of critical importance for the major sectors: health, environment, and agriculture to evolve creative scientific mechanisms to stem the deleterious effects of environmental pollution on the ecosystem. The pressing desire for a collaborative intersectoral approach in dealing with contemporary environmental disorders are only being gradually recognized globally, with the developing countries in particular, lagging in several respects.

### 7.1 Suggested Roles of the Agricultural, Health and Other Sectors in Combating Environmental Pollution From Aquatic and Terrestrial Derivation

The vital importance of agriculture and food production as part of environmental development imperative is well known. The role of the agricultural and related sectors is to mitigate the deleterious effects of ecological and environmental disorders that may be aquatic or terrestrial, by modifying food production with a bias for food crops that have counteractive effects on the adverse effects of pollution such as fruits and vegetables, with special attention to areas of environmental pollution and ecological shifts. This is probably why the Chinese with high incidence of cancer of the oesophagus and other parts of the gastrointestinal tract are currently among the greatest producers and consumers of fruit and vegetables (He et al., 2016; Fu et al., 2020; Wang et al., 2022). This may also be true of the Mediterranean diet in Europe with its health beneficial impact. While the current interest of the health sector is advocating increased consumption (Sorenson et al., 1999), that of the agricultural and the environmental sectors should be increased production under controlled conducive environment, necessitating ready availability of safe and protective nutrients or foods.

There has been an increasing awareness of the need to give priority to meeting the basic needs of people with emphasis on food security as well as health and education to enhance capacities for sustainable development. However, in the last few decades there have been trends that give rise to deep and continuing concern. Globally increasing environmental pollution and ecological disorders (Shifts) pose newer challenges that in addition to meeting basic food and health needs, the health, agricultural and environmental safety sectors must collaborate to jointly combat the effect of pollution and attendant health problems, which indeed are aspects of meeting health needs underpinned by restoration of altered physiology. This is probably in line with the early call by the 1992 Earth Summit for education for an environmentally sustainable future (Smyth, 2002).

The health sector has a responsibility to facilitate the understanding of the effect of pollution on aquatic and terrestrial components of the environment as well as the consequences on human health and advise the agricultural and environmental policy makers on strategies to ameliorate these attendant disorders. The progressively stronger scientific and clinical recognition of nutrition and diet to health implies the need for education in different aspects of nutrient (Anetor et al., 2006). In the United States, the collaboration between the Agriculture and Health sectors in this regard is worthy of emulation and extrapolation particularly in the developing World. This kind of partnership may help to curb a number of chronic diseases emanating from environmental pollution affecting both the aquatic and terrestrial components considerably through the pathway of oxidative stress.

## 8 Conclusion

There is substantial evidence that environmental pollution increases oxidative stress which affects aquatic and terrestrial organisms with far reaching pathological implications on human health. Pragmatically, mitigating pollutants associated oxidative stress may require a three-prong approach: bioremediation, which involves cleaning a polluted site by using naturally existing or intentionally introduced microbes to absorb and break down environmental contaminants, health education on disciplined environmental behavior as well as employing the principles of nutritional medicine essentially based on antioxidants derived mainly from plants. Dietary antioxidant supplementation, antioxidant micronutrients and increased consumption of fruit and vegetable may all help to neutralize or buffer the effects of oxidizing pollutants.

Understanding of the global picture of oxidative stress as well as integrating both the terrestrial and aquatic effects of environmental pollutants should be considered central to sustainable health of the population. Integrating this concept with health education and health promotion as a creative intervention, appears unarguably instructive and a worthy strategy that will aid in the prevention of disease and improvement of the quality of human life based on recognition of the pivotal role of oxidative stress and its mitigation by means of antioxidant approaches strongly premised on the antioxidant hypothesis.

## References

[B1] Agency for Toxic Substances and Disease Registry (ATSDR). (2016). Addendum to the Toxicological Profile for Arsenic. Availabe at: https://www.atsdr.cdc.gov/toxprofiles/Arsenic_addendum.pdf . (Accessed 31 January 2022) 37184170

[B2] Akram R. Natasha S. Fahad S. Hashmi M. Z. Wahid A. Adnan M. (2019). Trends of Electronic Waste Pollution and its Impact on the Global Environment and Ecosystem. Environ. Sci. Pollut. Res. 26 (17), 16923–16938. 10.1007/s11356-019-04998-2 31025281

[B3] Alagawany M. Elnesr S. S. Farag M. R. El-Naggar K. Madkour M. (2022). Nutrigenomics and Nutrigenetics in Poultry Nutrition: An Updated Review. World's Poult. Sci. J. 78 (2), 377–396. 10.1080/00439339.2022.2014288

[B4] Ali M. U. Siyi L. Yousaf B. Abbas Q. Hameed R. Zheng C. (2021). Emission Sources and Full Spectrum of Health Impacts of Black Carbon Associated Polycyclic Aromatic Hydrocarbons (PAHs) in Urban Environment: A Review. Crit. Rev. Environ. Sci. Technol. 51 (9), 857–896. 10.1080/10643389.2020.1738854

[B5] Ali W. Junaid M. Aslam M. W. Ali K. Rasool A. Zhang H. (2019). A Review on the Status of Mercury Pollution in Pakistan: Sources and Impacts. Arch. Environ. Contam. Toxicol. 76 (4), 519–527. 10.1007/s00244-019-00613-0 30915486

[B6] Almetwally A. A. Bin-Jumah M. Allam A. A. (2020). Ambient Air Pollution and its Influence on Human Health and Welfare: an Overview. Environ. Sci. Pollut. Res. 27 (20), 24815–24830. 10.1007/s11356-020-09042-2 32363462

[B7] Álvarez R. Vaz B. Gronemeyer H. de Lera Á. R. (2014). Functions, Therapeutic Applications, and Synthesis of Retinoids and Carotenoids. Chem. Rev. 114 (1), 1–125. 10.1021/cr400126u 24266866

[B8] Andreoli V. Sprovieri F. (2017). Genetic Aspects of Susceptibility to Mercury Toxicity: an Overview. Int. J. Environ. Res. Public Health 14 (1), 93. 10.3390/ijerph14010093 PMC5295343 28106810

[B9] Anetor J. Babalola O. Anetor G. (2006). Antioxidant Micronutrients as Intersectoral Link between Health and Agriculture. Afr J Biomed Res. 9 (1), 1–10. 10.4314/ajbr.v9i1.48766

[B10] Anetor J. I. Anetor G. O. Iyanda A. A. Adeniyi F. (2008). Environmental Chemicals and Human Neurotoxicity: Magnitude, Prognosis and Markers. Afr. J. Biomed. Res. 11, 1–12. 10.4314/ajbr.v11i1.50675

[B11] Bao X. Tian X. Yang C. Li Y. Hu Y. (2019). Association between Ambient Air Pollution and Hospital Admission for Epilepsy in Eastern China. Epilepsy Res. 152, 52–58. 10.1016/j.eplepsyres.2019.02.012 30909052

[B12] Baruah P. (2020). Effect of Soil Pollution on Humans, Plants and Animals. Available at: https://planningtank.com/environment/effect-soil-pollution-humans-plants-animals

[B13] Basu N. Horvat M. Evers D. C. Zastenskaya I. Weihe P. Tempowski J. (2018). A State-Of-The-Science Review of Mercury Biomarkers in Human Populations Worldwide between 2000 and 2018. Environ. Health Perspect. 126 (10), 106001. 10.1289/EHP3904 30407086 PMC6371716

[B14] Beckers F. Rinklebe J. (2017). Cycling of Mercury in the Environment: Sources, Fate, and Human Health Implications: A Review. Crit. Rev. Environ. Sci. Technol. 47 (9), 693–794. 10.1080/10643389.2017.1326277

[B15] Bello-Medina P. C. Rodríguez-Martínez E. Prado-Alcalá R. A. Rivas-Arancibia S. (2022). Ozone Pollution, Oxidative Stress, Synaptic Plasticity, and Neurodegeneration. Neurol. Engl. Ed. 37 (4), 277–286. 10.1016/j.nrleng.2018.10.025 34531154

[B16] Bertero A. Chiari M. Vitale N. Zanoni M. Faggionato E. Biancardi A. (2020). Types of Pesticides Involved in Domestic and Wild Animal Poisoning in Italy. Sci. Total Environ. 707, 136129. 10.1016/j.scitotenv.2019.136129 31869614

[B17] Buha Djordjevic A. Antonijevic E. Curcic M. Milovanovic V. Antonijevic B. (2020). Endocrine-disrupting Mechanisms of Polychlorinated Biphenyls. Curr. Opin. Toxicol. 19, 42–49. 10.1016/j.cotox.2019.10.006

[B18] Bundschuh J. Schneider J. Alam M. A. Niazi N. K. Herath I. Parvez F. (2021). Seven Potential Sources of Arsenic Pollution in Latin America and Their Environmental and Health Impacts. Sci. Total Environ. 780, 146274. 10.1016/j.scitotenv.2021.146274 34030289

[B19] Burk R. F. Hill K. E. (2015). Regulation of Selenium Metabolism and Transport. Annu. Rev. Nutr. 35, 109–134. 10.1146/annurev-nutr-071714-034250 25974694

[B20] Cai Y. Zhang B. Ke W. Feng B. Lin H. Xiao J. (2016). Associations of Short-Term and Long-Term Exposure to Ambient Air Pollutants with Hypertension. Hypertension 68 (1), 62–70. 10.1161/hypertensionaha.116.07218 27245182

[B21] Cakmak S. Dales R. E. Vidal C. B. (2010). Air Pollution and Hospitalization for Epilepsy in Chile. Environ. Int. 36, 501–505. 10.1016/j.envint.2010.03.008 20452673

[B22] Canipari R. De Santis L. Cecconi S. (2020). Female Fertility and Environmental Pollution. Int. J. Environ. Res. Public Health 17 (23), 8802. 10.3390/ijerph17238802 PMC7730072 33256215

[B23] Cao Y. Chen M. Dong D. Xie S. Liu M. (2020). Environmental Pollutants Damage Airway Epithelial Cell Cilia: Implications for the Prevention of Obstructive Lung Diseases. Thorac. Cancer 11 (3), 505–510. 10.1111/1759-7714.13323 31975505 PMC7049516

[B24] Carson R. (2002). Silent Spring. New York: First Mariner Books edition Houghton Miflin Company.

[B25] Catalani S. Donato F. Tomasi C. Pira E. Apostoli P. Boffetta P. (2019). Occupational and Environmental Exposure to Polychlorinated Biphenyls and Risk of Non-Hodgkin Lymphoma: A Systematic Review and Meta-Analysis of Epidemiology Studies. Eur. J. Cancer Prev. 28 (5), 441–450. 10.1097/cej.0000000000000463 30234686

[B26] Chang S.-S. Lin C.-Y. Lee M.-B. Shen L.-J. Gunnell D. Eddleston M. (2022). The Early Impact of Paraquat Ban on Suicide in Taiwan. Clin. Toxicol. 60 (1), 131–135. 10.1080/15563650.2021.1937642 34152240

[B27] Chang T. Y. Graff Zivin J. Gross T. Neidell M. (2019). The Effect of Pollution on Worker Productivity: Evidence from Call Center Workers in China. Am. Econ. J. Appl. Econ. 11, 151–172. 10.1257/app.20160436

[B28] Charkiewicz A. E. Backstrand J. R. (2020). Lead Toxicity and Pollution in Poland. Int. J. Environ. Res. Public Health 17 (12), 4385. 10.3390/ijerph17124385 PMC7345175 32570851

[B29] Chen Q.-H. Wu B.-K. Pan D. Sang L.-X. Chang B. (2021). Beta-carotene and its Protective Effect on Gastric Cancer. World J. Clin. Cases 9 (23), 6591–6607. 10.12998/wjcc.v9.i23.6591 34447808 PMC8362528

[B30] Chun J. Lee J. Ye L. Exler J. Eitenmiller R. R. (2006). Tocopherol and Tocotrienol Contents of Raw and Processed Fruits and Vegetables in the United States Diet. J. Food Compos. Analysis 19, 196–204. 10.1016/j.jfca.2005.08.001

[B31] Coffman A. H. (2018). “Rachel Carson: The Right Person, at the Right Time, with the Right Message,” in The Posthumous Nobel Prize in Chemistry. Ladies in Waiting for the Nobel Prize (Washington: American Chemical Society), 2, 217–243. 10.1021/bk-2018-1311.ch009

[B32] Coker E. Kizito S. (2018). A Narrative Review on the Human Health Effects of Ambient Air Pollution in Sub-Saharan Africa: An Urgent Need for Health Effects Studies. Int. J. Environ. Res. Public Health 15 (3), 427. 10.3390/ijerph15030427 PMC5876972 29494501

[B33] Cui L. Conway G. A. Jin L. Zhou J. Zhang J. Li X. (2017). Increase in Medical Emergency Calls and Calls for Central Nervous System Symptoms during a Severe Air Pollution Event, January 2013, Jinan City, China. Epidemiology 28 Suppl 1, S67–S73. 10.1097/EDE.0000000000000739 29028678

[B34] Das A. Sethi N. (2022). Modelling the Environmental Pollution-Institutional Quality Nexus in Low- and Middle-Income Countries: Exploring the Role of Financial Development and Educational Level. Environ. Dev. Sustain. 10.1007/s10668-021-02105-5

[B35] De Vos C. H. R. Vonk M. J. Vooijs R. Schat H. (1992). Glutathione Depletion Due to Copper-Induced Phytochelatin Synthesis Causes Oxidative Stress in Silene Cucubalus. Plant Physiol. 98 (3), 853–858. 10.1104/pp.98.3.853 16668756 PMC1080279

[B36] Devaki S. J. Raveendran R. L. (2017), Vitamin C: Sources, Functions, Sensing and Analysis. 10.5772/intechopen.70162 Available from: https://www.intechopen.com/chapters/56440

[B37] Durante M. Lenucci M. S. D'Amico L. D’Amico G. Mita G. (2014). Effect of Drying and Co-matrix Addition on the Yield and Quality of Supercritical CO2 Extracted Pumpkin (Cucurbita Moschata Duch.) Oil. Food Chem. 148, 314–320. 10.1016/j.foodchem.2013.10.051 24262563

[B38] Eddleston M. (2020). Poisoning by Pesticides. Medicine 48 (3), 214–217. 10.1016/j.mpmed.2019.12.019

[B39] Eftekhari A. Dizaj S. M. Chodari L. Sunar S. Hasanzadeh A. Ahmadian E. (2018). The Promising Future of Nano-Antioxidant Therapy against Environmental Pollutants Induced-Toxicities. Biomed. Pharmacother. 103, 1018–1027. 10.1016/j.biopha.2018.04.126 29710659

[B40] Ferrante M. C. Monnolo A. Del Piano F. Mattace Raso G. Meli R. (2022). The Pressing Issue of Micro- and Nanoplastic Contamination: Profiling the Reproductive Alterations Mediated by Oxidative Stress. Antioxidants 11 (2), 193. 10.3390/antiox11020193 35204076 PMC8868557

[B41] Flohé L. (2020). Looking Back at the Early Stages of Redox Biology. Antioxidants 9 (12), 1254. 10.3390/antiox9121254 PMC7763103 33317108

[B42] Forman H. J. Zhang H. (2021). Targeting Oxidative Stress in Disease: Promise and Limitations of Antioxidant Therapy. Nat. Rev. Drug Discov. 20 (9), 689–709. 10.1038/s41573-021-00233-1 34194012 PMC8243062

[B43] Fu X. Wang Q. Kuang H. Pinghui J. (2020). Mechanism of Chinese Medicinal-Medicated Leaven for Preventing and Treating Gastrointestinal Tract Diseases. Digestion 101 (6), 659–666. 10.1159/000493424 32980836

[B44] Fujiki Y. Bassik M. C. (2021). A New Paradigm in Catalase Research. Trends Cell Biol. 31 (3), 148–151. 10.1016/j.tcb.2020.12.006 33422360

[B45] Gaetke L. M. Chow C. K. (2003). Copper Toxicity, Oxidative Stress, and Antioxidant Nutrients. Toxicology 189 (1-2), 147–163. 10.1016/s0300-483x(03)00159-8 12821289

[B46] Gawel J. E. Ahner B. A. Friedland A. J. Morel F. M. M. (1996). Role for Heavy Metals in Forest Decline Indicated by Phytochelatin Measurements. Nature 381, 64–65. 10.1038/381064a0

[B47] Genchi G. Sinicropi M. S. Lauria G. Carocci A. Catalano A. (2020). The Effects of Cadmium Toxicity. Int. J. Environ. Res. Public Health 17 (11), 3782. 10.3390/ijerph17113782 PMC7312803 32466586

[B48] Gong W. Kong Y. (2022). Nonlinear Influence of Chinese Real Estate Development on Environmental Pollution: New Evidence from Spatial Econometric Model. Int. J. Environ. Res. Public Health 19 (1), 588. 10.3390/ijerph19010588 35010856 PMC8744668

[B49] Grill E. (1987). Phytochelatins, the Heavy Metal Binding Peptides of Plants: Characterization and Sequence Determination. Experientia 52, 317–322. 10.1007/978-3-0348-6784-9_28 2959522

[B50] Grill E. Winnacker E.-L. Zenk M. H. (1988). Occurrence of Heavy Metal Binding Phytochelatins in Plants Growing in a Mining Refuse Area. Experientia 44 (6), 539–540. 10.1007/bf01958944

[B51] Halliwell B. Gutteridge J. M. (2015). Free Radicals in Biology and Medicine. USA: Oxford University Press.

[B52] Hassaan M. A. El Nemr A. Hassaan M. A. El Nemr A. (2020). Pesticides Pollution: Classifications, Human Health Impact, Extraction and Treatment Techniques. Egypt. J. Aquatic Res. 46 (3), 207–220. 10.1016/j.ejar.2020.08.007

[B53] He Q.-L. Zhang L. Liu S.-Z. (2021). Effects of Polychlorinated Biphenyls on Animal Reproductive Systems and Epigenetic Modifications. Bull. Environ. Contam. Toxicol. 107 (3), 398–405. 10.1007/s00128-021-03285-6 34110444

[B54] He Y. Zhao L. Yu D. Fang H. Yu W. Guo Q. (2016). Consumption of Fruits and Vegetables in Chinese Adults from 2010 to 2012. Zhonghua Yu Fang. Yi Xue Za Zhi 50 (3), 221–224. 10.3760/cma.j.issn.0253-9624.2016.03.006 26957238

[B55] Helmenstine A. M. (2020). Free Radical Definition. Aug. 28, 2020. NY: ThoughtCo. Available at: thoughtco.com/definition-of-free-radical-604468

[B56] Hu H. (2019). Framework for Adding Environmental Exposure-Outcome Pairs to the Global Burden of Disease. Environ. Epidemiol. 3, 166. 10.1097/01.EE9.0000607608.03899.df

[B57] Hu W. Zhao C. Hu H. Yin S. (2021). Food Sources of Selenium and its Relationship with Chronic Diseases. Nutrients 13 (5), 1739. 10.3390/nu13051739 34065478 PMC8160805

[B58] Ighodaro O. M. Akinloye O. A. (2018). First Line Defence Antioxidants-Superoxide Dismutase (SOD), Catalase (CAT) and Glutathione Peroxidase (GPX): Their Fundamental Role in the Entire Antioxidant Defence Grid. Alexandria J. Med. 54 (4), 287–293. 10.1016/j.ajme.2017.09.001

[B59] Ikeh-Tawari E. Anetor J. Charles-Davies M. (2013). Cadmium Level in Pregnancy, Influence on Neonatal Birth Weight and Possible Amelioration by Some Essential Trace Elements. Toxicol. Int. 20 (1), 108–112. 10.4103/0971-6580.111558 23833446 PMC3702118

[B60] Irato P. Santovito G. (2021). Enzymatic and Non-enzymatic Molecules with Antioxidant Function. Antioxidants 10 (4), 579. 10.3390/antiox10040579 33918542 PMC8070535

[B61] Irimie A. Braicu C. Pasca S. Magdo L. Gulei D. Cojocneanu R. (2019). Role of Key Micronutrients from Nutrigenetic and Nutrigenomic Perspectives in Cancer Prevention. Medicina 55 (6), 283. 10.3390/medicina55060283 PMC6630934 31216637

[B152] Janghorbani M. Momeni F. Mansourian M. (2014). Systematic Review and Meta Analysis of Air Pollution Exposure and Risk of Diabetes. Eur. J. Epidemiol. 29:231–242. 10.1007/s10654-014-9907-2 24791705

[B62] Jia Z.-Q. Zhang Y.-C. Huang Q.-T. Jones A. K. Han Z.-J. Zhao C.-Q. (2020). Acute Toxicity, Bioconcentration, Elimination, Action Mode and Detoxification Metabolism of Broflanilide in Zebrafish, *Danio rerio* . J. Hazard. Mater. 394, 122521. 10.1016/j.jhazmat.2020.122521 32279005

[B63] Jiang Q. (2014). Natural Forms of Vitamin E: Metabolism, Antioxidant, and Anti-inflammatory Activities and Their Role in Disease Prevention and Therapy. Free Radic. Biol. Med. 72, 76–90. 10.1016/j.freeradbiomed.2014.03.035 24704972 PMC4120831

[B64] Kaput J. Rodriguez R. L. (2004). Nutritional Genomics: The Next Frontier in the Postgenomic Era. Physiol. Genomics 16 (2), 166–177. 10.1152/physiolgenomics.00107.2003 14726599

[B65] Kelly F. J. (2003). Oxidative Stress: Its Role in Air Pollution and Adverse Health Effects. Occup. Environ. Med. 60 (8), 612–616. 10.1136/oem.60.8.612 12883027 PMC1740593

[B66] Khosravi A. Bahonar A. Saadatnia M. Khorvash F. Maracy M. (2017). Carotenoids as Potential Antioxidant Agents in Stroke Prevention: A Systematic Review. Int. J. Prev. Med. 8, 70. 10.4103/ijpvm.IJPVM_112_17 28983399 PMC5625359

[B67] Landrigan P. J. Fuller R. Acosta N. J. R. Adeyi O. Arnold R. Basu N. (2018). The Lancet Commission on Pollution and Health. lancet 391 (10119), 462–512. 10.1016/s0140-6736(17)32345-0 29056410

[B68] Leni Z. Künzi L. Geiser M. (2020). Air Pollution Causing Oxidative Stress. Curr. Opin. Toxicol. 20-21, 1–8. 10.1016/j.cotox.2020.02.006

[B69] Liang W. Yang M. (2019). Urbanization, Economic Growth and Environmental Pollution: Evidence from China. Sustain. Comput. Inf. Syst. 21, 1–9. 10.1016/j.suscom.2018.11.007

[B70] Lindshield B. (2019). “Antioxidant Micronutrients,” in Human Nutrition (Oregon: Oregon state University). Availale at: https://open.oregonstate.education/humannutrition/chapter/antioxidant-micronutrients/ .

[B71] Ma J. Betts N. M. (2000). Zinc and Copper Intakes and Their Major Food Sources for Older Adults in the 1994-96 Continuing Survey of Food Intakes by Individuals (CSFII). J. Nutr. 130 (11), 2838–2843. 10.1093/jn/130.11.2838 11053529

[B72] Madamanchi N. R. Hausladen A. Alscher R. G. Amundson R. G. Fellows S. (1991). Seasonal Changes in Antioxidants in Red Spruce ( Picea Rubens Sarg.) from Three Field Sites in the Northeastern United States. New Phytol. 118 (2), 331–338. 10.1111/j.1469-8137.1991.tb00985.x 33874180

[B73] Malik A. Bagchi A. K. Vinayak K. Akolkar G. Slezak J. Belló-Klein A. (2021). Vitamin C: Historical Perspectives and Heart Failure. Heart Fail Rev. 26 (3), 699–709. 10.1007/s10741-020-10036-y 33033908

[B74] Maresca V. Bellini E. Landi S. Capasso G. Cianciullo P. Carraturo F. (2022). Biological Responses to Heavy Metal Stress in the Moss Leptodictyum Riparium (Hedw.) Warnst. Ecotoxicol. Environ. Saf. 229, 113078. 10.1016/j.ecoenv.2021.113078 34929502

[B75] Margaritelis N. V. Chatzinikolaou P. N. Chatzinikolaou A. N. Paschalis V. Theodorou A. A. Vrabas I. S. (2022). The Redox Signal: A Physiological Perspective. IUBMB life 74 (1), 29–40. 10.1002/iub.2550 34477294

[B76] Massányi P. Massányi M. Madeddu R. Stawarz R. Lukáč N. (2020). Effects of Cadmium, Lead, and Mercury on the Structure and Function of Reproductive Organs. Toxics 8 (4), 94. 10.3390/toxics8040094 PMC7711607 33137881

[B77] Mezynska M. Brzóska M. M. (2018). Environmental Exposure to Cadmium-A Risk for Health of the General Population in Industrialized Countries and Preventive Strategies. Environ. Sci. Pollut. Res. 25, 3211–3232. 10.1007/s11356-017-0827-z 29230653

[B78] Mikolajczyk S. Warenik-Bany M. Maszewski S. Pajurek M. (2020). Dioxins and PCBs - Environment Impact on Freshwater Fish Contamination and Risk to Consumers. Environ. Pollut. 263, 114611. 10.1016/j.envpol.2020.114611

[B79] Mojiri A. Zhou J. L. Ohashi A. Ozaki N. Kindaichi T. (2019). Comprehensive Review of Polycyclic Aromatic Hydrocarbons in Water Sources, Their Effects and Treatments. Sci. Total Environ. 696, 133971. 10.1016/j.scitotenv.2019.133971 31470323

[B80] Mortensen M. E. Wong L.-Y. Osterloh J. D. (2011). Smoking Status and Urine Cadmium above Levels Associated with Subclinical Renal Effects in U.S. Adults without Chronic Kidney Disease. Int. J. Hyg. Environ. Health 214, 305–310. 10.1016/j.ijheh.2011.03.004 21555245

[B81] Muñoz X. Barreiro E. Bustamante V. Lopez-Campos J. L. González-Barcala F. J. Cruz M. J. (2019). Diesel Exhausts Particles: Their Role in Increasing the Incidence of Asthma. Reviewing the Evidence of a Causal Link. Sci. Total Environ. 652, 1129–1138. 10.1016/j.scitotenv.2018.10.188 30586799

[B82] Murata K. Karita K. (2022). “Minamata Disease,” in Overcoming Environmental Risks to Achieve Sustainable Development Goals. Curr Topics Environ Health Prev Med (Singapore: Springer), 9–19. 10.1007/978-981-16-6249-2_2

[B83] Nhung N. T. T. Amini H. Schindler C. Kutlar Joss M. Dien T. M. Probst-Hensch N. (2017). Short-term Association between Ambient Air Pollution and Pneumonia in Children: A Systematic Review and Meta-Analysis of Time-Series and Case-Crossover Studies. Environ. Pollut. 230, 1000–1008. 10.1016/j.envpol.2017.07.063 28763933

[B84] Niki E. Noguchi N. (2021). Antioxidant Action of Vitamin E *In Vivo* as Assessed from its Reaction Products with Multiple Biological Oxidants. Free Radic. Res. 55 (4), 1–33. 10.1080/10715762.2020.1866181 33327809

[B85] Niño-savala A. G. Zhuang Z. Ma X. Fangmeier A. Li H. Tang A. (2019). Cadmium Pollution from Phosphate Fertilizers in Arable Soils and Crops: An Overview. Front. Agr. Sci. Eng. 6, 419–430. 10.15302/j-fase-2019273

[B86] Nwobi N. L. Adedapo S. K. Olukolade O. Oyinlade O. A. Lagunju I. A. Atulomah N. O. (2019). Positive and Inverse Correlation of Blood Lead Level with Erythrocyte Acetylcholinesterase and Intelligence Quotient in Children: Implications for Neurotoxicity. Interdiscip. Toxicol. 12 (3), 136–142. 10.2478/intox-2019-0016 32210702 PMC7085300

[B87] Nwobi N. L. Nwobi J. C. Adejumo E. N. Usiobeigbe O. S. Adetunji O. A. Atulomah N. O. (2021). Blood Lead Levels, Calcium Metabolism and Bone-Turnover Among Automobile Technicians in Sagamu, Nigeria: Implications for Elevated Risk of Susceptibility to Bone Diseases. Toxicol. Ind. Health 37 (11), 705–713. 10.1177/07482337211048963 34645326

[B88] Nwobi N. L. Nwobi J. C. Akinosun M. O. Atulomah N. O. Nwazuoke I. A. Anetor J. I. (2020). Impaired Antioxidant-Defence Status in Nigerian Children with Elevated Blood Lead Levels: A Possible Predisposing Factor to Chronic Diseases. J. Krishna Inst. Med. Sci. Univ. 9 (2), 1–8.

[B89] Obeng-Gyasi E. (2019). Sources of Lead Exposure in Various Countries. Rev. Environ. Health 34 (1), 25–34. 10.1515/reveh-2018-0037 30854835

[B90] Odo D. B. Yang I. A. Dey S. Hammer M. S. van Donkelaar A. Martin R. V. (2022). Ambient Air Pollution and Acute Respiratory Infection in Children Aged under 5 Years Living in 35 Developing Countries. Environ. Int. 159, 107019. 10.1016/j.envint.2021.107019 34875446

[B91] Olza J. Aranceta-Bartrina J. González-Gross M. Ortega R. Serra-Majem L. Varela-Moreiras G. (2017). Reported Dietary Intake and Food Sources of Zinc, Selenium, and Vitamins A, E and C in the Spanish Population: Findings from the ANIBES Study. Nutrients 9 (7), 697. 10.3390/nu9070697 PMC5537812 28684689

[B92] O’Rourke J. A. McCabe C. E. Graham M. A. (2020). Dynamic Gene Expression Changes in Response to Micronutrient, Macronutrient, and Multiple Stress Exposures in Soybean. Funct. Integr. Genomics 20 (3), 321–341. 31655948 10.1007/s10142-019-00709-9 PMC7152590

[B93] Owens K. Feldman J. Kepner J. (2010). Wide Range of Diseases Linked to Pesticides, Database Supports Policy Shift from Risk to Alternatives Assessment. Pesticides and You 30 (2), 13–21.

[B94] Pain D. J. Mateo R. Green R. E. (2019). Effects of Lead from Ammunition on Birds and Other Wildlife: A Review and Update. Ambio 48 (9), 935–953. 10.1007/s13280-019-01159-0 30879267 PMC6675766

[B95] Paithankar J. G. Saini S. Dwivedi S. Sharma A. Chowdhuri D. K. (2021). Heavy Metal Associated Health Hazards: An Interplay of Oxidative Stress and Signal Transduction. Chemosphere 262, 128350. 10.1016/j.chemosphere.2020.128350 33182141

[B96] Patel A. B. Shaikh S. Jain K. R. Desai C. Madamwar D. (2020). Polycyclic Aromatic Hydrocarbons: Sources, Toxicity, and Remediation Approaches. Front. Microbiol. 11, 562813. 10.3389/fmicb.2020.562813 33224110 PMC7674206

[B97] Patil P. V. Gendley M. K. Patil M. K. Prusti S. Doneria R. (2022). Applications of Nutrigenomics in Animal Health, Production and Reproduction. Pharma Innov. SP-11 (4), 524–527.

[B98] Patisaul H. B. (2021). Reproductive Toxicology: Endocrine Disruption and Reproductive Disorders: Impacts on Sexually Dimorphic Neuroendocrine Pathways. Reproduction 162 (5), F111–F130. 10.1530/rep-20-0596 33929341 PMC8484365

[B99] Pizzino G. Irrera N. Cucinotta M. Pallio G. Mannino F. Arcoraci V. (2017). Oxidative Stress: Harms and Benefits for Human Health. Oxidative Med. Cell. Longev. 2017, 8416763. 10.1155/2017/8416763 PMC5551541 28819546

[B100] Poljšak B. Fink R. (2014). The Protective Role of Antioxidants in the Defence against ROS/RNS-Mediated Environmental Pollution. Oxidative Med. Cell. Longev. 2014, 671539. 10.1155/2014/671539 PMC4129148 25140198

[B101] Prasad A. S. (2014). Zinc: An Antioxidant and Anti-inflammatory Agent: Role of Zinc in Degenerative Disorders of Aging. J. Trace Elem. Med. Biol. 28 (4), 364–371. 10.1016/j.jtemb.2014.07.019 25200490

[B102] Priya K. Setty M. Babu U. V. Pai K. S. R. (2021). Implications of Environmental Toxicants on Ovarian Follicles: How it Can Adversely Affect the Female Fertility? Environ. Sci. Pollut. Res. 28 (48), 67925–67939. 10.1007/s11356-021-16489-4 PMC8718383 34628616

[B103] Radwan M. A. El-Gendy K. S. Gad A. F. (2020). Biomarker Responses in Terrestrial Gastropods Exposed to Pollutants: A Comprehensive Review. Chemosphere 257, 127218. 10.1016/j.chemosphere.2020.127218 32497833

[B104] Rahaman M. S. Rahman M. M. Mise N. Sikder M. T. Ichihara G. Uddin M. K. (2021). Environmental Arsenic Exposure and its Contribution to Human Diseases, Toxicity Mechanism and Management. Environ. Pollut. 289, 117940. 10.1016/j.envpol.2021.117940 34426183

[B105] Raju N. J. (2022). Arsenic in the Geo-Environment: A Review of Sources, Geochemical Processes, Toxicity and Removal Technologies. Environ. Res. 203, 111782. 10.1016/j.envres.2021.111782 34343549

[B106] Ravindra K. Sokhi R. Vangrieken R. (2008). Atmospheric Polycyclic Aromatic Hydrocarbons: Source Attribution, Emission Factors and Regulation. Atmos. Environ. 42, 2895–2921. 10.1016/j.atmosenv.2007.12.010

[B107] Rehman K. Fatima F. Waheed I. Akash M. S. H. (2018). Prevalence of Exposure of Heavy Metals and Their Impact on Health Consequences. J. Cell. Biochem. 119, 157–184. 10.1002/jcb.26234 28643849

[B108] Rehman S. Usman Z. Rehman S. AlDraihem M. Rehman N. Rehman I. (2018). Endocrine Disrupting Chemicals and Impact on Male Reproductive Health. Transl. Androl. Urol. 7 (3), 490–503. 10.21037/tau.2018.05.17 30050807 PMC6043754

[B109] Reis L. d. P. G. Lora-Benítez A. J. Molina-López A. M. Mora-Medina R. Ayala-Soldado N. Moyano-Salvago M. d. R. (2022). Evaluation of the Toxicity of Bisphenol A in Reproduction and its Effect on Fertility and Embryonic Development in the Zebrafish (*Danio rerio*). Int. J. Environ. Res. Public Health 19 (2), 962. 10.3390/ijerph19020962 35055782 PMC8775542

[B110] Robaire B. Delbes G. Head J. A. Marlatt V. L. Martyniuk C. J. Reynaud S. (2022). A Cross-Species Comparative Approach to Assessing Multi- and Transgenerational Effects of Endocrine Disrupting Chemicals. Environ. Res. 204, 112063. 10.1016/j.envres.2021.112063 34562476

[B111] Rodrigues-Costa M. Fernandes M. S. d. S. Jurema-Santos G. C. Gonçalves L. V. d. P. Andrade-da-Costa B. L. d. S. (2021). Nutrigenomics in Parkinson's Disease: Diversity of Modulatory Actions of Polyphenols on Epigenetic Effects Induced by Toxins. Nutr. Neurosci., 1–13. 10.1080/1028415X.2021.2017662 36625764

[B112] Rodriguez-Amaya D. B. (2016). Structures and Analysis of Carotenoid Molecules. Subcell. Biochem. 79, 71–108. 10.1007/978-3-319-39126-7_3 27485219

[B113] Rosa A. C. Corsi D. Cavi N. Bruni N. Dosio F. (2021). Superoxide Dismutase Administration: A Review of Proposed Human Uses. Molecules 26 (7), 1844. 10.3390/molecules26071844 33805942 PMC8037464

[B114] Ruano C. S. M. Miralles F. Méhats C. Vaiman D. (2022). The Impact of Oxidative Stress of Environmental Origin on the Onset of Placental Diseases. Antioxidants 11 (1), 106. 10.3390/antiox11010106 35052610 PMC8773163

[B115] Saito T. Whatmore P. Taylor J. F. Fernandes J. M. O. Adam A. C. Tocher D. R. (2022). Micronutrient Supplementation Affects DNA Methylation in Male Gonads with Potential Intergenerational Epigenetic Inheritance Involving the Embryonic Development through Glutamate Receptor-Associated Genes. BMC genomics 23 (1), 115–118. 10.1186/s12864-022-08348-4 35144563 PMC8832813

[B116] Sandys O. Te Velde A. (2022). Raising the Alarm: Environmental Factors in the Onset and Maintenance of Chronic (Low-Grade) Inflammation in the Gastrointestinal Tract. Dig. Dis. Sci. 10.1007/s10620-021-07327-1 34981314

[B117] Schat H. Kalff M. M. A. (1992). Are Phytochelatins Involved in Differential Metal Tolerance or Do They Merely Reflect Metal-Imposed Strain? Plant Physiol. 99, 1475–1480. 10.1104/pp.99.4.1475 16669061 PMC1080650

[B118] Seiyaboh E. I. Izah S. C. (2019). Impacts of Soil Pollution on Air Quality under Nigerian Setting. J. Soil Water Sci. 3 (1), 45–53. 10.36959/624/430

[B119] Selin N. E. (2009). Global Biogeochemical Cycling of Mercury: A Review. Annu. Rev. Environ. Resour. 34, 43–63. 10.1146/annurev.environ.051308.084314

[B120] Shah A. S. Langrish J. P. Nair H. McAllister D. A. Hunter A. L. Donaldson K. (2013). Global Association of Air Pollution and Heart Failure: A Systematic Review and Meta-Analysis. Lancet 382 (9897), 1039–1048. 10.1016/s0140-6736(13)60898-3 23849322 PMC3809511

[B121] Shakeri M. Oskoueian E. Le H. H. Shakeri M. (2020). Strategies to Combat Heat Stress in Broiler Chickens: Unveiling the Roles of Selenium, Vitamin E and Vitamin C. Vet. Sci. 7, 1–9. 10.3390/vetsci7020071 PMC7356496 32492802

[B122] Shakman R. A. (1974). Nutritional Influences on the Toxicity of Environmental Pollutants. Archives Environ. Health Int. J. 28, 105–113. 10.1080/00039896.1974.10666447 4589147

[B123] Sharifi-Rad M. Anil Kumar N. V. Zucca P. Varoni E. M. Dini L. Panzarini E. (2020). Lifestyle, Oxidative Stress, and Antioxidants: Back and Forth in the Pathophysiology of Chronic Diseases. Front. Physiol. 11, 694. 10.3389/fphys.2020.00694 32714204 PMC7347016

[B124] Sideris L. H. Moore K. D. (2008). Introduction. Rachel Carson: Legacy and Challenge. Editors Sideris L. H. Moore K. D. (Albany: State University of New York Press).

[B125] Sirohi D. Al Ramadhani R. Knibbs L. D. (2021). Environmental Exposures to Endocrine Disrupting Chemicals (EDCs) and Their Role in Endometriosis: a Systematic Literature Review. Rev. Environ. Health 36 (1), 101–115. 10.1515/reveh-2020-0046 32903210

[B127] Smyth J. C., (2002). Are Educators Ready for the Next Earth Summit. Millennium Papers Series, 6. Available at: http://unitingnation.com/wp-content/uploads/2013/08/Educator_Readiness.pdf

[B128] Sorensen G. Stoddard A. Peterson K. Cohen N. Hunt M. K. Stein E. (1999). Increasing Fruit and Vegetable Consumption through Worksites and Families in the Treatwell 5-A-Day Study. Am. J. Public Health 89 (1), 54–60. 10.2105/ajph.89.1.54 9987465 PMC1508509

[B129] Souder W. (2012). On a Farther Shore: The Life and Legacy of Rachel Carson. New York: Crown Publishers. Available at: https://awionline.org/awi-quarterly/2012-fall/farther-shore-life-and-legacy-rachel-carson .

[B130] Steenland K. Barry V. Anttila A. Sallmen M. Mueller W. Ritchie P. (2019). Cancer Incidence Among Workers with Blood Lead Measurements in Two Countries. Occup. Environ. Med. 76, 603–610. 10.1136/oemed-2019-105786 31296664

[B131] Strange R. C. Spiteri M. A. Ramachandran S. Fryer A. A. (2001). Glutathione-S-transferase Family of Enzymes. Mutat. Res. 482 (1-2), 21–26. 10.1016/s0027-5107(01)00206-8 11535245

[B132] Sumanasekera W. K. Beckmann T. Fuller L. Castle M. Huff M. (2018). Epidemiology of Ovarian Cancer: Risk Factors and Prevention. Biomed. J Sci. Tech. Res. 11 (2), 1–13. 10.26717/BJSTR.2018.11.002076

[B134] Tinkov A. A. Filippini T. Ajsuvakova O. P. Skalnaya M. G. Aaseth J. Bjørklund G. (2018). Cadmium and Atherosclerosis: A Review of Toxicological Mechanisms and a Meta-Analysis of Epidemiologic Studies. Environ. Res. 162, 240–260. 10.1016/j.envres.2018.01.008 29358116

[B135] Traber M. G. Atkinson J. (2007). Vitamin E, Antioxidant and Nothing More. Free Radic. Biol. Med. 43, 4–15. 10.1016/j.freeradbiomed.2007.03.024 17561088 PMC2040110

[B136] Traina G. Bolzacchini E. Bonini M. Contini D. Mantecca P. Caimmi S. M. E. (2022). Role of Air Pollutants Mediated Oxidative Stress in Respiratory Diseases. Pediatr. Allergy Immunol. 33, 38–40. 10.1111/pai.13625 35080317 PMC9303668

[B137] Tripathy A. P. Dixit P. K. Panigrahi A. K. (2022). Impact of Effluent of Pulp & Paper Industry on the Flora of River Basin at Jaykaypur, Odisha, India and its Ecological Implications. Environ. Res. 204, 111769. 10.1016/j.envres.2021.111769 34419471

[B138] Tsai H.-J. Wu P.-Y. Huang J.-C. Chen S.-C. (2021). Environmental Pollution and Chronic Kidney Disease. Int. J. Med. Sci. 18 (5), 1121–1129. 10.7150/ijms.51594 33526971 PMC7847614

[B139] Vandana M. Priyadarshanee M. Mahto U. Das S. (2022). “Mechanism of Toxicity and Adverse Health Effects of Environmental Pollutants,” in Microbial Biodegradation and Bioremediation (Netherlands: Elsevier), 33–53. 10.1016/B978-0-323-85455-9.00024-2

[B140] Vesilind P. A. Peirce J. J. Weiner R. F. (2013). Environmental Pollution and Control. Netherland: Elsevier.

[B141] Wang J. Liu F. Li J. Huang K. Yang X. Chen J. (2022). Fruit and Vegetable Consumption, Cardiovascular Disease, and All-Cause Mortality in China. Sci. China Life Sci. 65 (1), 119–128. 10.1007/s11427-020-1896-x 33893978

[B142] Warra A. A. Prasad M. N. V. (2020). “African Perspective of Chemical Usage in Agriculture and Horticulture-Their Impact on Human Health and Environment,” in Agrochemicals Detection, Treatment and Remediation (Oxford: Butterworth-Heinemann), 401–436. 10.1016/B978-0-08-103017-2.00016-7

[B143] World Health Organization (WHO) (1997). Health and Environment in Sustainable Development: Five Years after the Earth Summit (No. WHO/EHG/97.8). Geneva: World Health Organization.

[B145] Xu X. Nie S. Ding H. Hou F. F. (2018). Environmental Pollution and Kidney Diseases. Nat. Rev. Nephrol. 14 (5), 313–324. 10.1038/nrneph.2018.11 29479079

[B146] You H. H. Song G. (2021). Review of Endocrine Disruptors on Male and Female Reproductive Systems. Comp. Biochem. Physiology Part C Toxicol. Pharmacol. 244, 109002. 10.1016/j.cbpc.2021.109002 33610819

[B147] Zaghloul A. Saber M. Gadow S. Awad F. (2020). Biological Indicators for Pollution Detection in Terrestrial and Aquatic Ecosystems. Bull. Natl. Res. Cent. 44 (1), 1–11. 10.1186/s42269-020-00385-x

[B148] Zandalinas S. I. Fritschi F. B. Mittler R. (2021). Global Warming, Climate Change, and Environmental Pollution: Recipe for a Multifactorial Stress Combination Disaster. Trends Plant Sci. 26 (6), 588–599. 10.1016/j.tplants.2021.02.011 33745784

[B149] Zhang H. Dong H. Ren M. Liang Q. Shen X. Wang Q. (2020). Ambient Air Pollution Exposure and Gestational Diabetes Mellitus in Guangzhou, China: a Prospective Cohort Study. Sci. Total Environ. 699, 134390. 10.1016/j.scitotenv.2019.134390 31525546

[B150] Zheng S. Yang Y. Wen C. Liu W. Cao L. Feng X. (2021). Effects of Environmental Contaminants in Water Resources on Nonalcoholic Fatty Liver Disease. Environ. Int. 154, 106555. 10.1016/j.envint.2021.106555 33857709

[B151] Zhitkovich A. (2021). Ascorbate: Antioxidant and Biochemical Activities and Their Importance for *In Vitro* Models. Arch. Toxicol. 95, 3623–3631. 10.1007/s00204-021-03167-0 34596731 PMC8541910

